# Soft plasma electrolysis with complex ions for optimizing electrochemical performance

**DOI:** 10.1038/srep44458

**Published:** 2017-03-10

**Authors:** Muhammad Prisla Kamil, Mosab Kaseem, Young Gun Ko

**Affiliations:** 1 School of Materials Science and Engineering, Yeungnam University, Gyeongsan 38541, Republic of Korea

## Abstract

Plasma electrolytic oxidation (PEO) was a promising surface treatment for light metals to tailor an oxide layer with excellent properties. However, porous coating structure was generally exhibited due to excessive plasma discharges, restraining its performance. The present work utilized ethylenediaminetetraacetic acid (EDTA) and Cu-EDTA complexing agents as electrolyte additives that alter the plasma discharges to improve the electrochemical properties of Al-1.1Mg alloy coated by PEO. To achieve this purpose, PEO coatings were fabricated under an alternating current in silicate electrolytes containing EDTA and Cu-EDTA. EDTA complexes were found to modify the plasma discharging behaviour during PEO that led to a lower porosity than that without additives. This was attributed to a more homogeneous electrical field throughout the PEO process while the coating growth would be maintained by an excess of dissolved Al due to the EDTA complexes. When Cu-EDTA was used, the number of discharge channels in the coating layer was lower than that with EDTA due to the incorporation of Cu_2_O and CuO altering the dielectric behaviour. Accordingly, the sample in the electrolyte containing Cu-EDTA constituted superior corrosion resistance to that with EDTA. The electrochemical mechanism for excellent corrosion protection was elucidated in the context of equivalent circuit model.

A plasma electrolytic oxidation (PEO) has been one of the novel electrochemistry-based surface treatments that utilized plasma state by applying a high voltage, allowing a hierarchy of metals and alloys such as Al, Mg, Ti, and Zr to form ceramic oxide coatings with excellent wear and corrosion resistance^1^,^2^,^3^. In contrast to conventional anodizing, PEO could be applied in an electrolyte solution with high pH, which rendered this method to be eco-friendly. Thus, PEO coating has been used in various applications of the electronic, automobile, and aircraft industries^4^,^5^.

To date, the development of PEO technique were intrigued by a trade-off in the role of plasma discharges. In one hand, they would be critical to the coating growth, allowing the oxide coating to reach a thickness of tens to hundreds of μm^1^,^2^,^3^, significantly higher than that of the natural passive layer provided by the bare alloys. On the other hand, the plasma discharges led to microstructural defects such as micro-pores and cracks through repeated cycle of rapid melting-solidification processes.

In order to suppress the detrimental effects of plasma discharges, significant attention was paid towards the development of soft sparking regime during PEO process^6^,^7^,^8^,^9^, which was associated with the intensity decrease of plasma discharges while maintaining the coating growth. The utilization of this regime, however, was limited in terms of practical application due to the need of a sophisticated voltage/current waveform with an extended coating time. For instance, Matykina *et al*.^6^ and Jaspard-Mécuson *et al*.^7^ reported using different adjustments of bipolar current that the soft sparking regime started to appear from ~20 and ~40 min of coating time, respectively. In addition, Aliashgari *et al*.^8^ and Arrabal *et al*.^9^ observed the soft sparking regime starting from ~10 min on Ti and Mg alloys, respectively by using a square waveform.

Thus, to decrease the defects in the PEO coating, a more simple approach would be desirable to conduct PEO with softer plasma. In this regard, electrolyte additives emerged as a potential candidate due to their apparent influence to the microstructure, composition and electrochemical behaviour of the resultant coating layer^10^,^11^,^12^. Among these additives, EDTA (ethylenediamine tetraacetic acid) was commonly used. For instance, Shi *et al*.^13^ compared the effects of EDTA and sodium tetraborate as additives within the silicate-phosphate electrolyte and they reported that the corrosion resistance of the coating with EDTA was superior due to the development of a compact coating structure. On the other hand, EDTA was capable of producing complex ions with transition metals, allowing easier incorporation of metal oxides into PEO coating due to the formation of the negatively-charged species. Rogov^14^ utilized such inherent properties of EDTA in order to incorporate several transition metals into the coating layers by reacting either metallic solid or salt with EDTA prior to PEO. Interestingly, the use of pre-reacted metal-EDTA in a complex salt form in PEO process has not been reported to date despite the potential for industrial application. In addition, although several studies have addressed the effect of EDTA, few discussions have been argued to date in terms of its role during PEO and the related mechanisms to form the compact coatings.

Therefore, this work aimed to tailor a less defective PEO coating by the addition of EDTA and metal-EDTA complexes which might modify plasma discharges. The roles of each additive were discussed in relation to the plasma characteristics and microstructural features. Pre-reacted metal-EDTA was used, contrary to the previous work^14^, to ensure the presence of the complex ion without an additional pH adjustment. Cu-EDTA was chosen for the metal-EDTA mainly due to the simple stoichiometry and the possible applications of copper oxides such as photovoltaic and gas sensor devices^15^,^16^. Table 1 lists the composition, pH, and conductivity of the electrolytes without additives, with EDTA, and with Cu-EDTA, which were referred to as Bath A, Bath B, and Bath C, respectively. Furthermore, the electrochemical performance of the PEO-coated samples related to corrosion-protection properties was evaluated in a 3.5 wt.% of NaCl to simulate the salinity level of typical seawater. The results were elaborated by taking the structure and composition of the ceramic layer into consideration.

## Results

### Plasma discharge characteristics

**Figure 1:**
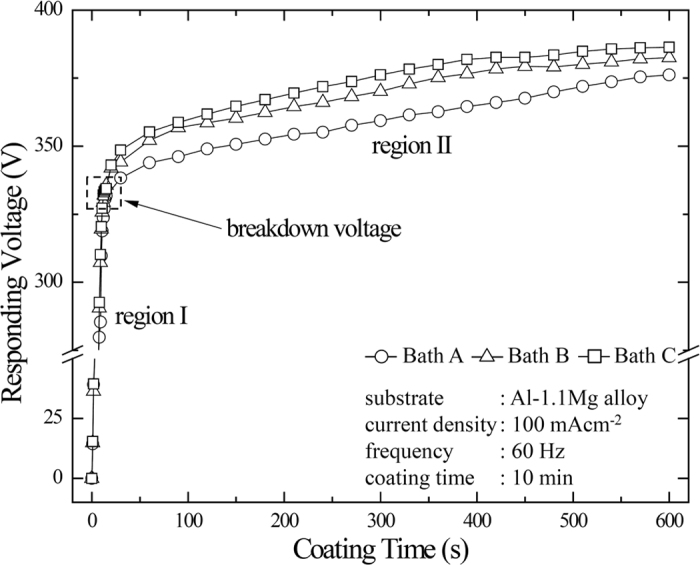
Responding voltage (rms) vs. coating time curves of the samples during PEO at 100 mAcm^−2^ utilizing different electrolyte conditions. Region I and II are divided by the rate of voltage increase and the occurrence of plasma discharges.

Figure 1 presents the rms voltage-time curves of the samples in Baths A, B, and C during PEO process for 10 min at a constant current density of 100 mAcm^−2^. Two regions of the curves would be identified as per the increasing trend of the responding voltages with respect to the coating time. Irrespective of the electrolytes, the rate of the voltage increase in region I was relatively higher than that in regions II. The sharp increase of the responding voltage in the early stages of PEO coating was attributed to an increase in electrical resistance which resulted from the formation of the thin passive film formed initially on substrate. Moreover, Na_2_SiO_3_ used in the present electrolytes would promote such metal passivation which enhanced an increase in the responding voltage^1^. Intense plasma discharges started to appear when the voltage reached a critical value that would be referred to as the sparking voltage. The plasma discharges in liquid medium would exhibit non-equilibrium behaviour where all species of plasma such as electrons, neutrals and ions have different temperatures^17^,^18^. Upon the mechanism of breakdown in PEO, a model was proposed by Hussein *et al*.^19^,^20^ by correlating the results of spectroscopic study on plasma discharges with the final structure of the oxide coating. In their model, breakdown mechanism was attributed presumably to (i) the gas discharges at coating-electrolyte interface for weak plasma discharge (or apparent micro-pores) and (ii) the dielectric breakdown of the oxide film for strong plasma discharge (or severe discharge channels). The model was consistent with the spectroscopic investigation by Jovovic *et al*.^21^. Once the plasma discharges took place, massive bubbles might appear and, thereby, affect the microstructure significantly. Same cases have been found in the articles reported by Cheng *et al*.^22^ and Troughton *et al*.^23^.

It was measured that the values of the sparking voltages remained constant in all conditions, reaching ~320 V in 20 sec owing to the identical electrolyte conductivity associated with the concentration of KOH as well as the occurrence of the initial passivation related to the concentration of Na_2_SiO_3_^2^,^24^. Interestingly, the final voltage increased in the order of Baths A, B, and C whose values were measured to be 376, 382, and 386 V. As reported earlier^12^,^25^, the difference in final voltages was be attributed to several factors such as the coating thickness, morphology, and coating composition. These factors would affect significantly the characteristics of plasma discharges such as their size, duration, density and energy^26^,^27^,^28^.

**Figure 2:**
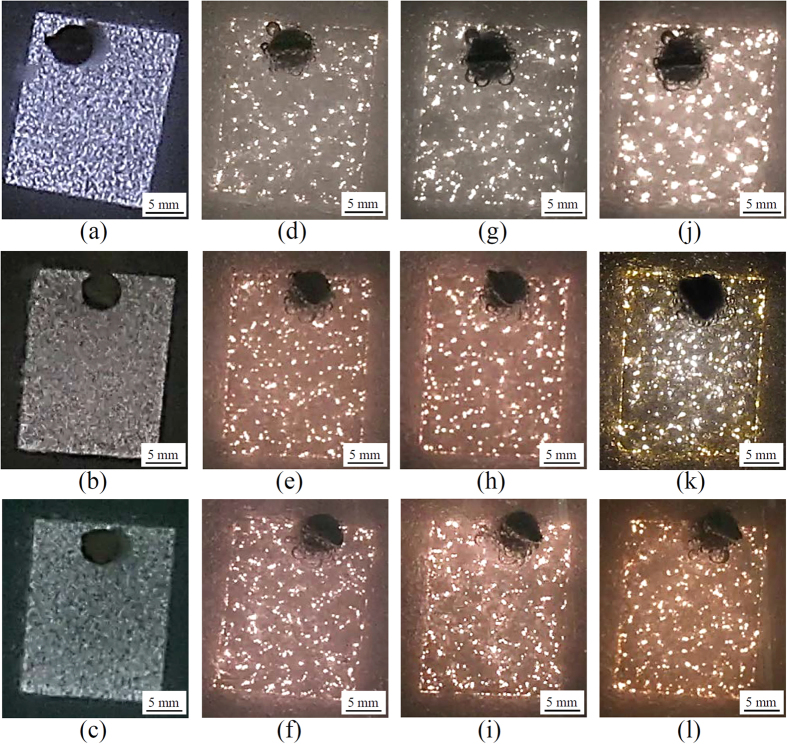
Optical images showing the appearance of plasma discharges during PEO at 100 mAcm^−2^ utilizing electrolyte conditions of Bath A, B, and C, respectively at different coating time of (**a,b,c**) 20 s, (**d,e,f**) 180 s, (**g,h,i**) 300 s, (**j,k,l**) 540 s. The plasma discharges first appeared at 20 s.

**Figure 3:**
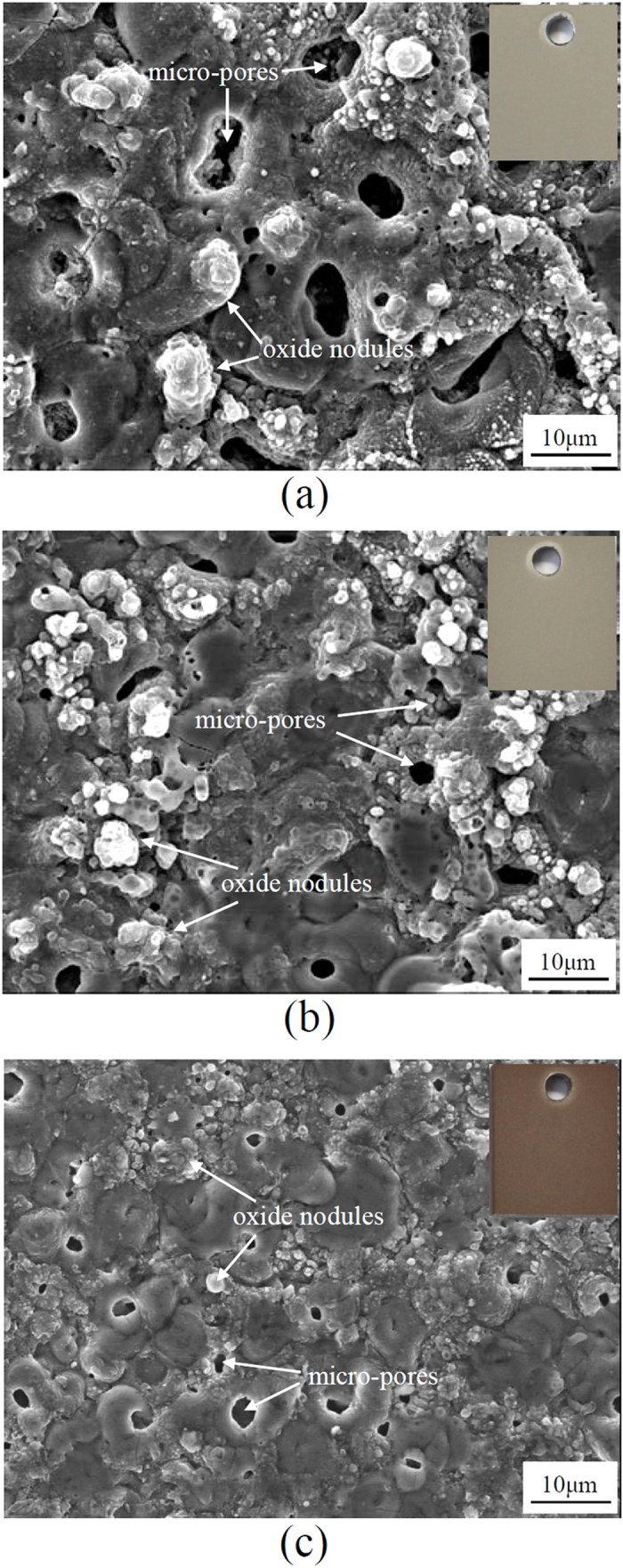
SEM images showing the surface morphologies of the samples treated by PEO at 100 mAcm^−2^ for 10 min using different electrolytes of (**a**) Bath A, (**b)** Bath B, and (**c**) Bath C. The inset figures show surface appearances of the respective conditions. The surfaces of oxide layers comprise the micro-pores and oxide nodules.

Figure 2 shows the appearances of plasma discharges at different time of the PEO process. Fig. 2(a,b) and (c) presents the appearances of the plasma discharges of three different samples at ~20 s when the sparking voltage was reached. At the onset of discharge activities, the plasma sparks appeared to be flashing homogeneously throughout the entire surface irrespective of the electrolyte conditions. Immediately after, their characteristics changed swiftly, forming a visible cascade of discharges at the sites with lowest resistance^27^,^29^, as shown in Fig. 2(d,e) and (f) for Bath A, B, and C, respectively at 180 s. At this stage, the density of the discharges in Bath A appeared to be lower and less homogeneous than Baths B and C. This spatial distributions continued for the majority of coating time, while the size of discharge cascades increased gradually, as indicated by Fig. 2(g,h,i) which were taken at 300 s. However, when the coating time reached ~540 s, the appearance of discharges of three different samples varied significantly. Figure 2(j) shows that the sample in Bath A exhibited bright, violent cascades whose size reached ~1 mm, which might be related to the dielectric breakdown of the oxide layer^17^,^18^,^19^. Such bright cascades were also found in Fig. 2(k) for Bath B, but with their size decreased to ~0.5 mm. In contrast, with the addition of Cu-EDTA in Bath C as shown in Fig. 2 (l), such violent discharges were not found. After PEO coatings were finished in this study, the coating colour of the samples using Baths A and B were medium grey apparently while the colour of the sample using Bath C looked as light brown, as seen from the photographs shown in the insets of Fig. 3. This fact implied the formation of some Cu-compounds in case of the electrolyte with Cu-EDTA.

### Morphologies of coating layer

Figure 3 displays the SEM images showing the surface morphologies of the coating layers formed in Baths A, B, and C. Irrespective of the electrolyte compositions used in this study, the surface morphologies comprised a number of ‘micrometre-sized pores and oxide nodules. The micro-pores were formed mainly by the activity of micro-discharges at the interface between the sample surface and electrolyte^19^,^30^. Although such formation of the micro-pores might lower efficiently the residual stress in the coating layer during PEO involving the melting and cooling processes together, they would provide a short-circuit path for easier infiltration of corrosion medium down to the inner region close to the substrate^12^,^31^,^32^. Thereafter, the overall endurance in corrosive environment might be deteriorated due to the change in local pH^33^ which caused a decrease in the energy barrier of the electrical double layer against electrochemical corrosion and triggered the dissolution of the oxide coating^34^. On the other hand, the formation of the oxide nodules was due mainly to the rapid solidification of molten oxides. Lee *et al*.^35^ estimated that, through an analysis of melting point and phase transition of different compounds, the temperature of the coating layer during PEO might reach approximately 2116–2643 K. As soon as the oxide melted, it was solidified with ease due to the rapid cooling by the electrolyte whose temperature was kept at 288 K during PEO coating.

From Fig. 3(a), it was suggested that the pore size and fraction of the coating layer in Bath A were approximately ~5 μm and ~7.0%, respectively. When the coating layer was fabricated in Bath B, as shown in Fig. 3(b), the average pore size and porosity was decreased to ~2.5 μm and ~2.5%. Interestingly, from Fig. 3(c) it was evident that the coating layer fabricated in Bath C exhibited the lowest average pore size and porosity of ~1.5 μm and ~1.2% respectively. This suggested a decrease in the detrimental effect of plasma discharges which might be attributed to the alteration of their size and distribution in the presence of EDTA complexes, as shown in Fig. 2. This result was consistent with earlier reports^13^,^36^ which demonstrated that the use of an EDTA-containing electrolyte in PEO led to more uniform and compact coating layers.

**Figure 4:**
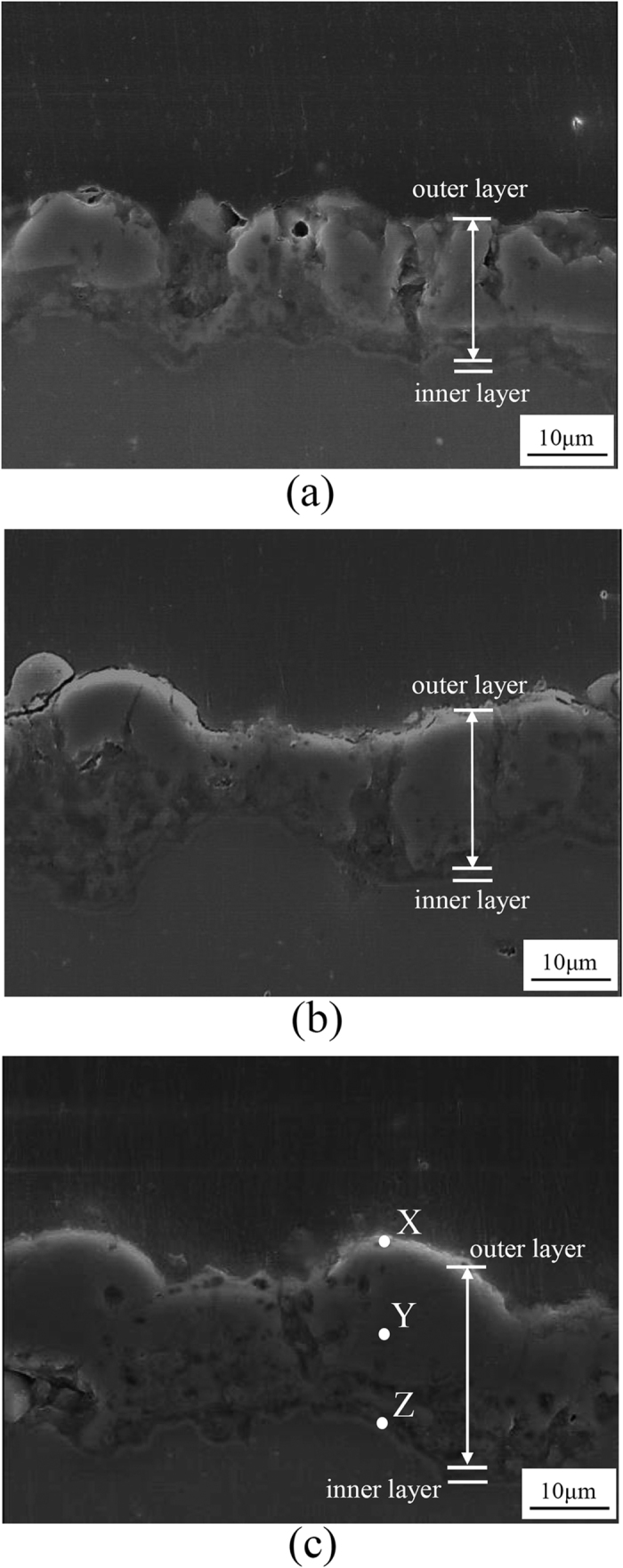
SEM images showing the cross-sectional morphologies of the samples treated by PEO at 100 mAcm^−2^ for 10 min using different electrolytes of (**a**) Bath A, (**b**) Bath B, and (**c**) Bath C. X, Y, and Z in (**c**) are representative points for EDS measurement. The oxide layers consist of the outer and inner layers exhibiting a number of discharge channels.

Figure 4 presents SEM images showing the cross-sectional area for all of the PEO-coated samples to provide more comprehensive insight of the coatings morphology. The coatings were composed of two distinct layers namely the outer layer and the inner layer. The inner layers were similar in terms of thickness regardless of the electrolytes. No cracks were observed in the vicinity of substrate-coating interface due to the sintered interlocking, implying good substrate-coating adhesion. The average thicknesses of the coating layers in Baths A, B and C, as shown in Fig. 4(a,b) and (c), were ~15 ± 2 μm, ~16 ± 3 μm and ~17 ± 2 μm, respectively, suggesting that the coating thickness of all PEO-coated samples were relatively identical even though the plasma discharges were suppressed in Baths B and C. The result suggested the occurrence of plasma discharges with decreased intensity without an expense of coating growth, i.e., soft plasma. Considering the similarity of *V-t* behaviour, the plasma softening in this study would depend solely on the roles of additives instead of the current regime and coating time as previously reported^6^,^7^,^8^,^9^.

More importantly, the coating layer of all samples showed a considerable difference in the number of discharge channels, which were generated by the dielectric breakdown of oxide layer initiated on structural defects that developed to localized melt channel perpendicular to the coating surface^19^,^30^,^37^. Figure 4(a) shows that the PEO-coated sample in Bath A exhibited the highest number of discharge channels. When the sample was treated in Baths B and C electrolytes, appreciable suppression of the strong discharges took place, resulting in the coating layers having low number of discharge channels (Fig. 4(b) and (c) for Baths B and C, respectively). This result implied that these channels might be associated with the bright and violent discharges observed in Fig. 2. The PEO-coated sample in Bath C appeared to comprise the lowest number of discharge channels which suggest a suppression of detrimental discharges due to the effect of Cu-compounds.

### Chemical composition of coating layer

**Figure 5:**
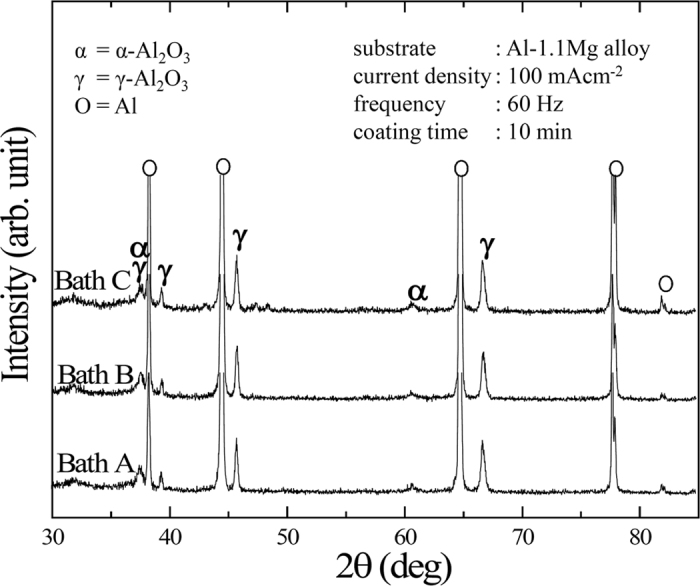
XRD spectra of the samples treated by PEO using Baths A, B, and C. The scan ranges are from 30 to 85^0^ with Cu Kα radiation source. The Al substrate, α-Al_2_O_3_, and γ-Al_2_O_3_ are identified.

Figure 5 presents XRD spectra of the coatings fabricated in all of the present electrolytes. The spectra were composed mainly of peaks associated with γ-Al_2_O_3_ and Al metal, together with minor peaks of α-Al_2_O_3_, regardless of the electrolytes. The Al metal peaks were attributed to the substrate, most likely because the penetration of X-ray radiation was deep enough to reach beyond the coating layers. According to an investigation by Levin and Brandon^38^, a typical sequence in phase transformation of anodic Al_2_O_3_ would be γ-δ-θ-α with respect to the increase in temperature. Despite the inherent fact that α-Al_2_O_3_ was the most stable phase of aluminium oxides, γ-Al_2_O_3_ was found to be the major component of PEO coatings under the present experimental conditions. This was attributed to the dominant influence of a rapid cooling rate induced by the electrolyte, preventing further transformation of the metastable phase such as γ-Al_2_O_3_ to other stable phases^31^. It was apparent from the XRD spectra in Baths B and C that no additional peak related to EDTA or other carbon-based compounds was detected. In addition, peaks from constitutive compounds containing Cu was unlikely to be identified, which might be attributed to the melting of the Cu-compounds due to the high temperature during PEO, deteriorating their crystallinity.

To confirm the Cu-compound in the coating fabricated under Bath C condition, an analysis of chemical elements was carried out via EDS. The EDS measurements were taken at several points from the outer, middle, and inner parts of the coating indicated by X, Y and Z in Fig. 4(c) as representative points. The results confirmed the incorporation of Cu-compound which was localized on the outer part of the coating, as listed in Table 2. Similar findings were also observed for Si, in which detection at the outer part was the highest, decreasing for the middle and inner part significantly. Previous works on PEO^22^,^39^,^40^ also reported that the incorporation of electrolyte species tend to take place easier in the outer part of the coating layer. This might be attributed to tendency to decrease the high surface energy at the outer part of the coating^41^, allowing incorporation of electrolyte species on the surface with ease during the repeated melting-solidification processes when the electrical double layer might be disrupted. Another possible factor affecting the extent of incorporation would be the size of electrolyte species^42^. The Cu-containing species in the electrolyte, especially [Cu-EDTA]^2–^ ions, were notably larger than the majority of other species (O^2−^, OH^−^, H_2_O), inhibiting their movement deeper through the coating. In case of Si, the element was possible to be incorporated in deeper parts of the coating considering the size of SiO_3_^2−^ ion was smaller and the concentration was significantly higher than [Cu-EDTA]^2−^.

**Figure 6:**
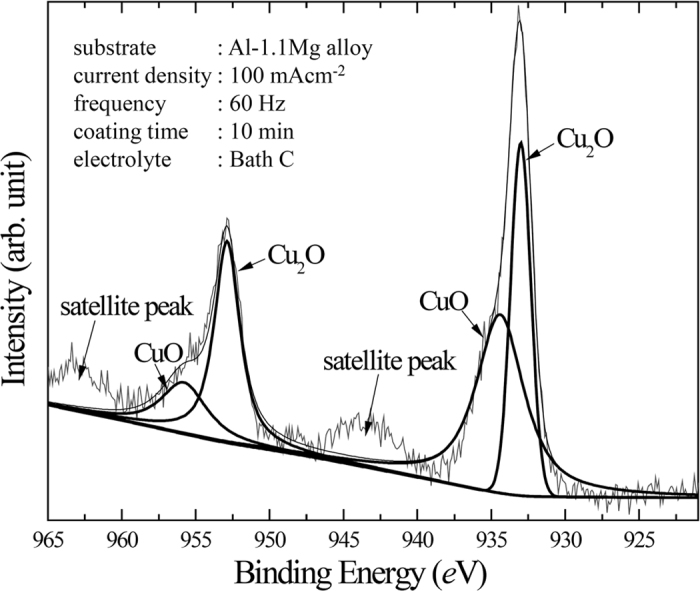
XPS spectra analysis of Cu 2*p* in the sample treated by PEO using Bath C. The deconvolutions imply the coexistence of Cu_2_O and CuO in the oxide layer. The satellite peaks are associated with the presence of CuO.

Figure 6 presents XPS spectrum for Cu 2*p* of the coating in Bath C, which was analysed to determine the identity of Cu-compound incorporated in the coating layer. Peak deconvolution was applied based on the following binding energies: 933.2 eV (2*p* 3/2) and 952.0 (2*p* 1/2) for Cu_2_O and 935.7 eV (2*p* 3/2) and 955.8 (2*p* 1/2 for CuO^43^. Moreover, peaks at 944.7 eV and 964.8 eV corresponded to the satellite peaks of CuO originating from the screening of 2*p* electrons by the that in the 3*d*^9^ orbital^44^,^45^. In the light of the XPS analysis, it was confirmed that both Cu_2_O and CuO were incorporated in the coating, where Cu_2_O was the more dominant compound, as indicated by the higher intensities of the deconvoluted peaks. Thus, the Cu_2_O content was believed to be responsible for the light brown colour of the coating layer in Bath C. The relative amounts of Cu_2_O over CuO were estimated on the basis of integrated peak intensities following equation (1),


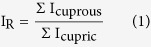


where *I*_*cuprous*_, *I*_*cupric*_, and *I*_*R*_ referred to integrated peak intensities of Cu_2_O, CuO, and the intensity ratio, respectively. The intensity ratio of Cu_2_O over CuO was found to be ~1.52, showing that the amount of Cu_2_O was superior to that of CuO. On the contrary, earlier studies^43^,^46^ had reported that CuO was more stable than Cu_2_O. This conflicting result of the present study might be attributed to the fact that, while the Cu_2_O would start to decompose at relatively low temperature (450 K)^47^, its transformation was suppressed by the rapid cooling provided by the bulk electrolyte, resulting in higher amount of Cu_2_O as compared to that of CuO.

### Electrochemical responses of the coating layer

**Figure 7:**
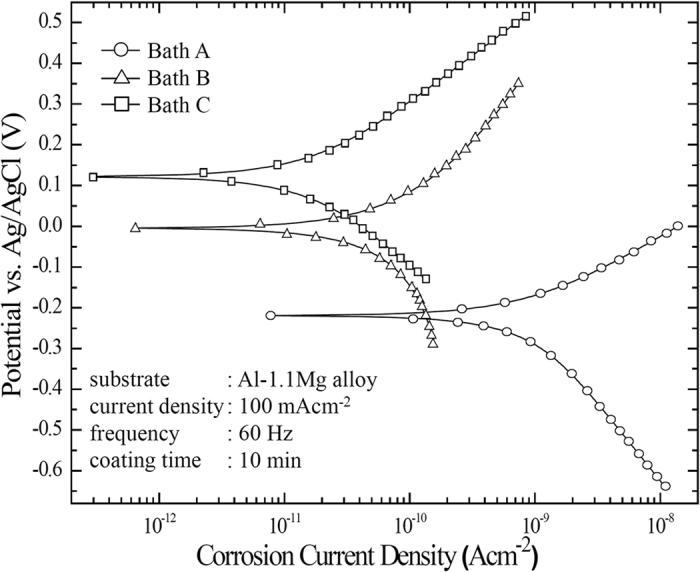
Potentiodynamic polarization curves of the samples treated by PEO using Baths A, B, and C, which are measured from −0.25 to 0.4 V vs. open circuit potential in 3.5 wt.% NaCl solution.

Electrochemical responses of the PEO-coated samples related to a corrosion process was evaluated by potentiodynamic polarization test and electrochemical impedance spectroscopy (EIS) in a 3.5 wt.% NaCl solution which would be similar to the salinity of sea water. The potentiodynamic polarization test measured from −0.25 V to 0.4 V vs. open circuit potential (OCP). Figure 7 presents the polarization curves of the PEO-coated samples fabricated under all electrolytes. The corrosion parameters from this test, namely the corrosion potential (*E*_*corr*_), corrosion current density (*i*_*corr*_) and Tafel slopes for anodic and cathodic branches (*β*_*a*_ and *β*_*c*_, respectively), were extracted from the polarization curve using Tafel extrapolation method, as listed in Table 3. The corrosion resistances of all samples were compared using their respective values of polarization resistance (*R*_*p*_), which were calculated based on the Stern-Geary equation shown in equation (2)^48^,^49^.


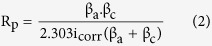


For the sample treated in Bath B containing EDTA, the polarization curve shifted to a lower region of current density and a nobler region of potential due to the lower coating porosity as compared to that treated in the basic electrolyte of Bath A. Similarly, when the sample was treated in Bath C, which contained Cu-EDTA instead of EDTA, the polarization curve exhibited further shifting to the aforementioned directions. Accordingly, the Tafel extrapolation suggested that the samples in Bath B and C had a successively lower *i*_*corr*_ and higher *E*_*corr*_ relative to that in Bath A. In general, the PEO-coated sample with low *i*_*corr*_ and/or high *E*_*corr*_ would exhibit excellent corrosion resistance^2^,^10^,^12^,^31^. Low value of *i*_*corr*_ indicated that the coating layer exhibited better resistance against the transportation of corrosive species, which was mainly attributed to the microstructural factor. In addition, high value of *E*_*corr*_, indicating a low tendency towards the oxidative reaction in the corrosive medium, would also be affected apparently by the composition of the coating layer.

**Figure 8:**
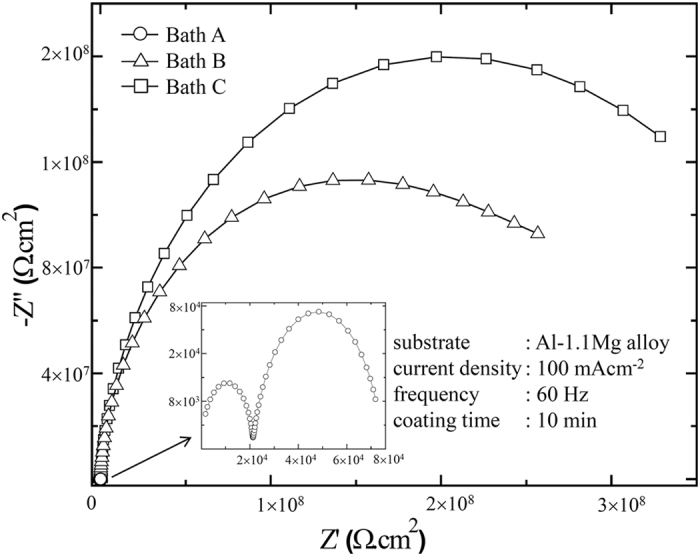
EIS Nyquist plots of the samples treated by PEO using different electrolytes of Bath A, B, and C, which are conducted from 10^6^ to 0.1 Hz in 3.5 wt.% NaCl solution. The inset shows the magnified spectra of the sample using Bath A.

To further investigate electrochemical response relating to the corrosion behaviour of the PEO-coated samples in all electrolyte conditions, EIS tests were conducted to reveal the mechanism of corrosion protection. Figure 8 presents the EIS spectra of the PEO-coated samples presented in the form of Nyquist plot ranging from 10^6^ to 0.1 Hz with 10 mV rms perturbation in a 3.5 wt.% NaCl solution as the corrosion medium. In general, the Nyquist plot of all the PEO-coated samples exhibited a semicircle-shaped arc associated with a combination of resistive and capacitive behaviour of the samples. In the Nyquist plot of EIS, the resistive behaviour would correspond to the real axis (*Z’*) while the capacitive behaviour would be represented by the imaginary axis (*-Z”*). As such, the complex impedance of the sample under investigation will be proportional to the diameter of the semicircle. From Fig. 8 it was evident that the complex impedance of the PEO-coated samples in Baths B and C were significantly higher than that in Bath A. It was also confirmed that the highest impedance was obtained by the sample in Bath C. This fact indicated that, as compared to other conditions in the present study, the coating in Bath C would provide superior protection for the metallic substrate against corrosion process, which was in agreement with the results from the potentiodynamic polarization test.

The inset in Fig. 8 showed that the Nyquist plot of the sample in Bath A consisted of two semicircles, implying two responses of different time constants. According to previous investigations in the field of PEO^9^,^10^,^12^,^31^, the time constant of high frequency would typically ascribed to the outer layer while that of low frequency was related to the response from the inner layer. In case for PEO-coated samples in Baths B and C, the two semicircles might overlap one another due to the concurrent increase in radius. The unfinished semicircles for Baths B and C might be associated with oxide layers with exceptionally high resistance^32^,^49^ which suggested that the coating layers treated in Baths B and C attained higher impedances to that treated in Bath A.

## Discussion

The formation of oxide layer during PEO in general would be a complex phenomenon due to the non-equilibrium behaviour of the plasma species^17^,^18^,^50^. In the vicinity of the plasma, these reactive species together with the massive amount of thermal energy might accelerate the electrochemical reactions significantly and allow metastable compounds or phases to form^35^,^51^,^52^. The plasma-activated electrochemical reactions responsible for the oxide formation involved two different mechanisms such as that via direct oxidation on the surface and that via dissolution of the substrate. A general electrochemical reaction for direct oxidation, as shown in equation (3), might involve solid Al substrate and oxygen-containing species in electrolyte such as H_2_O. On the other hand, the oxide layer formation via substrate dissolution, as shown in equation (4), might involve Al ions that were ejected from the substrate as a result of electron transfer, and the abundant hydroxide ions. A subsequent dehydration reaction might take place provided the high temperature of the plasma discharges as shown in equation (5), thus forming the resultant oxide.


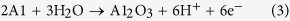



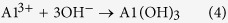



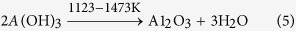


Although both mechanisms would take place simultaneously, the species existed in the electrolyte might provide favourable condition for one particular mechanism to prevail. According to the earlier report by Treacy *et al*.^53^, an addition of EDTA would promote the anodic dissolution of Al metal in an aqueous solution by forming soluble complex ion, as shown in equation (6). This effect would be even more pronounced in an alkaline solution due to full deprotonation of EDTA, stabilizing the EDTA^4–^ ions. In a conventional anodic oxidation process, the dissolution of substrate was unfavourable since it would act as a competitor against the oxide formation. Interestingly, due to the plasma-assisted reactions in PEO process, the Al species originated from substrate dissolution might also contribute to the formation of the coating layer, as shown inequation (7), followed by a dehydration reaction depicted in equation (5) to form Al_2_O_3_.


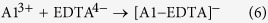



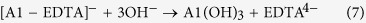


The decrease in porosity level of the coating layer fabricated in electrolyte containing EDTA-based ions might be related to the influence of the intermediate Al(OH)_3_ species via substrate dissolution. Previous investigations^54^,^55^ demonstrated that the Al(OH)_3_ formation from Al in aqueous solution would result in an amorphous-colloidal form. Hsu *et al*.^55^ reported that such Al(OH)_3_ formation would impede the high electric field between the anode and electrolyte, inhibiting the generation of the plasma discharges. In addition, the presence of EDTA was reported to exhibit buffering action^53^ which might cause the following effects: (i) stabilization of the electrical double layer, which was important to maintain plasma discharges^56^,^57^ and (ii) inhibition to the structural breakdown of the oxide layer, leading to a more homogeneous electrical field distribution throughout PEO process. Thus, lower porosity of the oxide layer would be achieved through a relatively sluggish yet homogeneous plasma discharges due to the alteration of electrical field. On the other hand, the coating growth would be maintained owing to the compensation by the abundant Al ions provided by EDTA and a stable electrical double layer. These synergistic effects might be responsible for soft plasma discharges formed during PEO process with EDTA-based ions.

It was important to note that, the stability of a complex ion comprising EDTA and a metal ion could be represented by its complex formation constant (*K*_*f*_). The complex formation constant involving an EDTA^4–^ ligand and a metal ion M^n+^ would be defined as in equation (8).


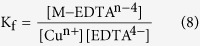


The value of log *K*_*f*_ for [Al-EDTA]^−^ and [Cu-EDTA]^2–^ complex ions were 16.1 and 18.8, respectively^58^, indicating that [Cu-EDTA]^2–^ would be more stable as compared to [Al-EDTA]^−^. Therefore, it might be assumed that the Al ions ejected from the substrate would not affect the movement of Cu towards the anode in the form of [Cu-EDTA]^2−^ ions.

Similar to the formation mechanism of Al_2_O_3_ discussed previously, in the vicinity of the substrate Cu-EDTA complex would first be converted to insoluble Cu-hydroxides, followed by thermal dehydration to form Cu oxides, as shown in equations (9) and (10).


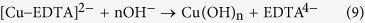



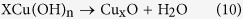


After the PEO process with EDTA and Cu-EDTA, no evidence relating to their incorporation was found in the coating layer due to the susceptibility of EDTA complexes to thermal decomposition under the high temperature of PEO. According to previous studies^59^,^60^, the decomposition of EDTA would result in several possible products: (i) ethylenediamine triacetic acid (ED3A) through a loss of the acetate group, (ii) N-(2-hydroxyethyl) iminodiacetic acid + iminodiacetic acid through a C-N bond cleavage, or (iii) CO_2_ through a complete decomposition. Apparently, the possible formation of CO_2_ appeared to exhibit no influence to the porosity of the samples in this study due to the low concentration of EDTA. This thermal decomposition process would occur in the vicinity of the plasma discharges adjacent to the surface, allowing the Cu bound in the Cu-EDTA complex to be incorporated in the coating layers by the above mechanism, regardless the decomposition of EDTA molecules.

From Fig.4(c), it was suggested that the occurrence of discharge channels was impeded apparently when the electrolyte contained Cu-EDTA. This phenomenon might be attributed to a change in dielectric behaviour of the coating layer due to the addition of Cu_2_O and CuO. The dielectric constant for Al_2_O_3_ was reported to be in the range of ~4.5 to ~9.3 from a thin film to bulk dimension^34^,^61^, whereas the dielectric constants for Cu_2_O and CuO could reach to magnitudes around ~12 and ~18.1, respectively^61^,^62^. In addition, local dielectric constant at the surfaces and interfaces between components was reported to be significantly higher than their corresponding bulk value^63^. Materials with a higher dielectric constant would be able to withstand a stronger electric field before reaching an electrical breakdown^64^. The existence of Cu oxides might lead to an inhibition of discharge channels, which was reported to be highly affected by composition of the coating layer instead of the electrolyte conditions^19^,^30^,^37^. Hence, it was suggested that the change in dielectric behaviour due to Cu_2_O/CuO and softening of plasma discharges by EDTA molecules provided a synergistic effect, resulting in the coating layer with low level of surface porosity and low number of discharge channels when an electrolyte containing Cu-EDTA was employed.

The results from potentiodynamic polarization and impedance spectroscopy (Figs 7 and 8, respectively) were in agreement to the fact that the PEO-coated sample in Bath C would provide an exceptional corrosion resistance as compared to that in other electrolytes. This might be attributed to three different factors, i.e., the morphology, composition, and thickness of the coating layer. First, superior morphological features of the sample coated in Bath C, such as low level of porosity and less number of discharge channels, would allow limited amount of the corrosion medium to infiltrate the coating layer. Second, Cu-oxides incorporated in the coating tended to affect the corrosion resistance by an increment of *E*_*corr*_ or impedance. Third, the coating thickness would also strongly affect the corrosion behaviour. However, since the coating thicknesses between the present samples were relatively constant, only the coating morphology and composition were considered to be the substantial factors affecting the corrosion resistance.

**Figure 9:**
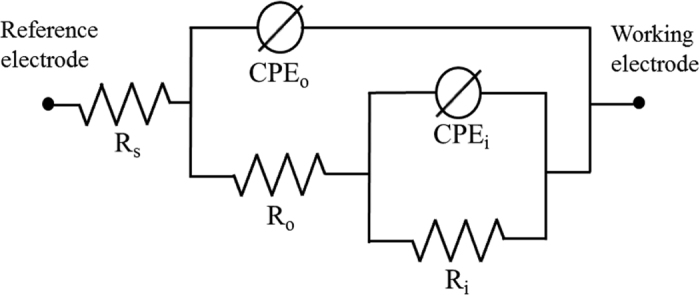
Equivalent circuit model used to analyze EIS Nyquist plots of the samples treated by PEO using Baths A, B, and C. The present model comprises the time constants reflecting electrochemical behaviour of the outer and inner layers.

To provide a more comprehensive display of the electrochemical behaviour, the data obtained via EIS was simulated by assigning an equivalent circuit model according to the electrical responses of the system under investigation^65^,^66^. Figure 9 presents the equivalent circuit model assigned for all of the present samples, from which the parameter values were obtained and listed in Table 4. In this equivalent circuit model, *R*_*s*_, *R*_*o*_ and *R*_*i*_ referred to the resistance of the interfacial electrolyte, the outer layer and the inner layer respectively. *CPE*_*o*_ and *CPE*_*i*_ referred to the constant phase element of outer and inner layer, respectively. The constant phase elements were used in preference to a capacitor in order to address the surface inhomogeneity factor resulting in a non-uniform distribution of current flow through the coating. CPE was defined by equation (11) as follows.


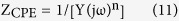


*Z*_*CPE*_ was the impedance value of the CPE, *j* was the imaginary number, *ω* was the angular frequency, *Y* and *n* was CPE parameters. Parameter *n,* whose value varied between 0 and 1, was related to the extent of the non-uniform current distribution, where a high value of *n* would imply good surface homogeneity^55^. A semicircle in the Nyquist plot obtained in this study would refer to a resistive and a capacitive response in parallel^10^,^31^,^67^. Hence, in Fig. 9, *R*_*o*_ and *CPE*_*o*_ were constructed in parallel, as well as for *R*_*i*_ and *CPE*_*i*_. Taking the physical properties of the system into consideration, the resistive behaviour could be associated with the insulating property of the ceramic coating which consisted mainly of Al_2_O_3_, while the capacitive behaviour could be associated with the double layer capacitance exhibited when the coating layer separated two conductive medium: the metal substrate and the electrolyte. Furthermore, the existence of the micro-pores and oxide nodules would be related with the resistance and parameter *n* of the CPE, where their values would be proportional to the homogeneity of the coating layer. By comparing *R*_*o*_ and *n*_*o*_ in Table 4, it was evident that the homogeneity of coating layer in Bath C would be superior due to smaller size and fraction and micro-pores and smaller oxide nodules, which might suggest a delayed porous transport of bulk corrosive medium into the coating layer as compared to that in other conditions.

The parallel combination of two R-C time constants representing inner and outer layers might indicated that no strict boundary existed between the two layers. Some of the existing discharge channels might provide short path for the corrosive medium to reach the inner layer, leading to a condition where a part of the inner and outer layers exhibited similar electrochemical properties, which could be indicated by overlapping semicircles in the Nyquist plot.

In view of a corrosion process in practical environment, the role of capacitance could be neglected since the corrosion process might not involve an external source of electrical current. Thus, the resistance was the only parameter considered to represent the corrosion properties of the coating layers. The *R*_*i*_ values of Baths B were approximately 4 orders of magnitude higher as compared to Bath A which mainly be responsible for the improvement of corrosion properties evaluated via potentiodynamic polarization. Interestingly, based on EIS measurements presented in Table 4, the *R*_*i*_ of PEO coatings fabricated with EDTA and Cu-EDTA were relatively similar. This suggested that the two additives might exhibit similar mechanism to affect the development of the inner layer due to the EDTA^4–^ ions produced by both additives. On the other hand, the *R*_*o*_ of the coating with Cu-EDTA was higher than that with EDTA due to the difference in porosity and the number of discharge channels in their outer layers, which would be responsible for the improvement of corrosion resistance via potentiodynamic polarization. Thus, it was suggested that both inner and outer layers contributed in the overall corrosion properties of the coating layer.

Upon the comparing the magnitude of resistances, the *R*_*i*_ values were superior to that of *R*_*o*_ for all conditions. The predominance of the inner layer over the outer layer in terms of resistance was also observed in earlier investigations of PEO coatings, for instance, Wen *et al*.^68^ on a 2024 Al alloy and Ghasemi *et al*.^69^ on an AM50 Mg alloy. In those studies, however, the difference in resistances between the outer and inner layers was only 1–2 orders of magnitude as compared to ~4 orders of magnitude reported in the present work by employing EDTA and Cu-EDTA, implying both additives played a significant role in improving the inner parts of the coating layer.

By the discussions presented above, it could be suggested that both EDTA and Cu-EDTA would promote the formation of soft plasma discharges similarly. Coating growth would be maintained by electrochemical reactions involving EDTA-based complex ions even though the plasma intensity would be decreased. In addition, the copper oxides formed in the coating layer with the addition of Cu-EDTA might impede the development of discharge channels throughout the oxide layer. This effect together with the plasma softening would be accounted for the superior microstructure and corrosion resistance of coating layer formed in electrolyte with Cu-EDTA. Further investigation utilizing optical emission spectroscopy would be needed to reveal the mechanisms of the plasma softening, ultimately aiming towards a compact coating layer with functional properties by using complex ions.

## Conclusions

The influence of EDTA complexes in the silicate electrolyte on the corrosion properties of Al-1.1Mg alloy sample via PEO was investigated in relation to the characteristics of the microstructure and plasma discharges. First, the additions of EDTA and Cu-EDTA into the present electrolyte were found to decrease the size and density of the discharge cascades, especially from ~540 s where stronger discharges would be evident. Thus, the open porosities decreased from ~7% to ~2.5% and ~1.2% for the coating layer with EDTA and Cu-EDTA, respectively. This was attributed to softer plasma discharges which appeared when a relatively sluggish yet homogeneous electrical field was generated together with an excess of Al ions in a stabilized electrical double layer that sustained the coating growth under a weaker field. On the other hand, the number of discharge channels throughout the coating with the addition of Cu-EDTA was lower as compared to that with EDTA, which was due mainly to an appreciable change in dielectric behaviour with the incorporations of Cu_2_O and CuO via plasma-assisted electrochemical reactions with Cu-EDTA. According to the present electrochemical analyses using potentiodynamic polarization and impedance spectroscopy tests in 3.5 wt.% NaCl solution, it is therefore that an excellent corrosion resistance would be attained by the ceramic coating layer formed in the electrolyte containing Cu-EDTA. The present results strongly suggested that the use of EDTA and metal-EDTA complexes as electrolyte additives would be a potential approach to reduce significantly the defects formed in PEO coating layer without the use of complicated current waveform and an extended coating time.

## Materials and Methods

An Al alloy plate with a chemical composition of 1.1 Mg, 0.71 Si, 0.5 Fe, 0.24 Cu, 0.19 Cr, 0.12 Mn, 0.05 Zn, 0.05 Ti, and balance Al (in wt.%) was cut into samples with a dimension of 30 mm (L) × 20 mm (W) × 5 mm (T). All samples were ground mechanically with SiC paper up to 1200 grit, rinsed with distilled water and cleaned ultrasonically with pure ethanol prior to PEO coating. The present sample was set as the anode while stainless steel net was used as the cathode. A glass vessel was used as the electrolyte container, equipped with magnetic stirrer and water cooling system to maintain the electrolyte temperature around 288 K for the stabilization of electrochemical reactions throughout the process. A series of PEO coating treatments were performed for 10 mins under an AC current with current density and frequency of 100 mAcm^−2^ and 60 Hz, respectively. The composition, pH, and conductivity of the electrolytes used in this study are listed in Table 1. The following reagent-grade chemicals were used to prepare the electrolytes: potassium hydroxide (KOH), sodium silicate (Na_2_SiO_3_), EDTA (Na_2_H_2_-EDTA), and Cu-EDTA (CuNa_2_-EDTA).

The surface and cross-sectional morphologies of the PEO-coated samples were observed via field-emission scanning electron microscopy (FE-SEM, Hitachi S-4100) coupled with an energy-dispersive X-ray spectroscopy (EDS, Horiba Inc.). All samples were coated with platinum prior to SEM observations to avoid charging effect by the electrons. The measurements of the size and fraction of micro-pores were performed by SEM observations taken from at least ten different areas for each condition by an aid of image analyser software. The 2-D direct calculation using SEM images was preferred over plausible 3-D methods reported previously^70^ because of two reasons that might affect the experimental result. First, the application of the above-mentioned techniques would likely to require the delamination of the coating layer from the substrate which would damage significantly the inner layer close to the substrate. Second, the porosity results utilizing the 3-D techniques might be exaggerated because they measure not only the micro-pores but also the oxide nodules. X-ray diffraction (XRD, Rigaku D/MAX-2500) was applied to analyse the constituent compounds and their phases in the ceramic layer. In addition, the chemical composition was analysed further by X-ray photoelectron spectroscopy (XPS, VG Microtech ESCA 2000). The electrochemical response of the PEO coatings relating to corrosion behaviour was investigated by a potentiodynamic polarization test and electrochemical impedance spectroscopy (EIS) in a 3.5 wt.% NaCl solution. No agitation was performed during both tests. The polarization curves were measured from −0.25 to 0.4 V with respect to the open circuit potential (OCP) at a scan rate of 1 mVs^−1^ whereas the EIS experiments were conducted from 10^6^ to 0.1 Hz at an interval of 10 points/decade with a 10 mV rms. Both electrochemical measurements were carried out in a potentiostat (Gamry Interface1000) utilizing a three-electrode cell system: the PEO-coated sample with an exposed area of 1 cm^2^ as the working electrode, a platinum plate as the counter electrode, and Ag/AgCl as the reference electrode.

## Additional Information

**How to cite this article:** Prisla Kamil, M. *et al*. Soft plasma electrolysis with complex ions for optimizing electrochemical performance. *Sci. Rep.* **7**, 44458; doi: 10.1038/srep44458 (2017).

**Publisher's note:** Springer Nature remains neutral with regard to jurisdictional claims in published maps and institutional affiliations.

## Figures and Tables

**Table 1:** Composition, pH, and conductivity of the electrolytes used for PEO coatings.

Electrolyte	Composition (gL^−1^)				pH	Conductivity (mScm^−1^)
	KOH	Na_2_SiO_3_	EDTA	Cu-EDTA		
Bath A	6	4	0	0	12.94	22.5
Bath B	6	4	1	0	12.90	22.3
Bath C	6	4	0	1	12.83	22.1

All measurements are conducted at 288 K which is identical to the PEO temperature.

**Table 2:** EDS results of the sample treated by PEO using Bath C.

Point	Al (wt.%)	O (wt.%)	Si (wt.%)	Cu (wt.%)
X	31.9 ± 2.0	51.8 ± 1.4	8.7 ± 1.2	7.6 ± 1.3
Y	45.1 ± 1.8	53.2 ± 1.1	1.7 ± 0.7	—
Z	45.3 ± 1.4	54.3 ± 1.2	0.4 ± 0.1	—

The measurements are taken from the outer, middle, and inner parts of the coating layers, which are denoted by points X, Y, and Z in Fig. 3(c).

**Table 3:** Potentiodynamic polarization results of the samples treated by PEO using different electrolytes measured from −0.25 to 0.4 V vs. open circuit potential in 3.5 wt.% NaCl solution. *E*_*corr*_, *I*_*corr*_, *β*_*a*_, and *β*_*c*_ are obtained by Tafel extrapolation.

Electrolyte	*E*_*corr*_ (mV)	*I*_*corr*_(Acm^−2^)	*β*_*a*_(V)	*β*_*c*_(V)	*R*_*p*_(Ωcm^2^)
Bath A	−219.6	8.1 × 10^−10^	1.8 × 10^−1^	3.9 × 10^−1^	6.6 × 10^7^
Bath B	−4.1	2.3 × 10^−10^	2.0 × 10^−1^	1.4 × 10^−1^	1.6 × 10^8^
Bath C	132.9	1.2 × 10^−11^	5.8 × 10^−1^	3.7 × 10^−1^	8.2 × 10^9^

*R*_*p*_is calculated based on the Stern-Geary equation.

**Table 4:** Electrochemical impedance parameters of the samples treated by PEO using different electrolytes measured from 10^6^ to 0.1 Hz in 3.5 wt.% NaCl solution.

Electrolyte	*R*_*s*_ (*Ωcm*^*2*^)	*R*_*o*_ (*Ωcm*^*2*^)	*n*_*o*_	*Y*_*o*_(*Ss*^*n*^*cm*^*−2*^)	*R*_*i*_(Ωcm^2^)	*n*_*i*_	*Y*_*i*_(*Ss*^*n*^*cm*^*−2*^)
Bath A	26.6	1.2 × 10^4^	0.86	3.9 × 10^−9^	5.4 × 10^4^	0.19	7.1 × 10^−6^
Bath B	23.7	3.8 × 10^5^	0.94	7.9 × 10^−10^	4.9 × 10^8^	0.84	1.1 × 10^−9^
Bath C	22.3	8.6 × 10^6^	0.99	2.1 × 10^−11^	8.6 × 10^8^	0.86	1.8 × 10^−9^
